# Editorial: Novel pathophysiologic mechanisms and reno-protective pharmacotherapies in diabetic kidney disease

**DOI:** 10.3389/fphar.2024.1531748

**Published:** 2024-12-11

**Authors:** Elias John Elenjickal, Anna T. Valson, Santosh Varughese, Lloyd Vincent, Edwin Fernando, Gopalakrishnan Natarajan

**Affiliations:** ^1^ Department of Nephrology, Christian Medical College and Hospital, Vellore, India; ^2^ Department of Renal Medicine, Aberdeen Royal Infirmary, Aberdeen, Scotland, United Kingdom; ^3^ The University of Melbourne, Parkville, VIC, Australia; ^4^ Africa Healthcare Network, Port Blair, Mauritius; ^5^ Department of Nephrology, Stanley Medical College, Chennai, Tamil Nadu, India; ^6^ Department of Nephrology, Madras Medical College, Chennai, Tamil Nadu, India

**Keywords:** diabetes mellitus, pathomechanisms, oxidative stress, SGLT 2 inhibitor, GLP-1 recepter agonist, astrgaloside IV, huangkui capsule, ophiocordyceps sinensis

Diabetic kidney disease (DKD) is a global health problem and is an important risk factor for kidney failure and cardiovascular (CV) disease (Alicic et al., 2017). Until recently, renin angiotensin-aldosterone system inhibitors (RAASi) were the only available agents with proven renoprotective benefit (Brenner et al., 2001; Lewis et al., 2001). In 2019, sodium glucose co-transporter-2 inhibitors (SGLT-2i) revolutionized the management of DKD with added cardiovascular and renal benefits (Perkovic et al., 2019; Heerspink et al., 2020; The EMPA-KIDNEY Collaborative Group et al., 2023). The last few years have been promising with the discovery of newer therapies in DKD targeting diverse pathophysiologic pathways (Heerspink et al., 2019; Bakris et al., 2020; Perkovic et al., 2024). Despite these existing therapies, there is a substantial residual risk of kidney disease progression (Figure 1).

**FIGURE 1:**
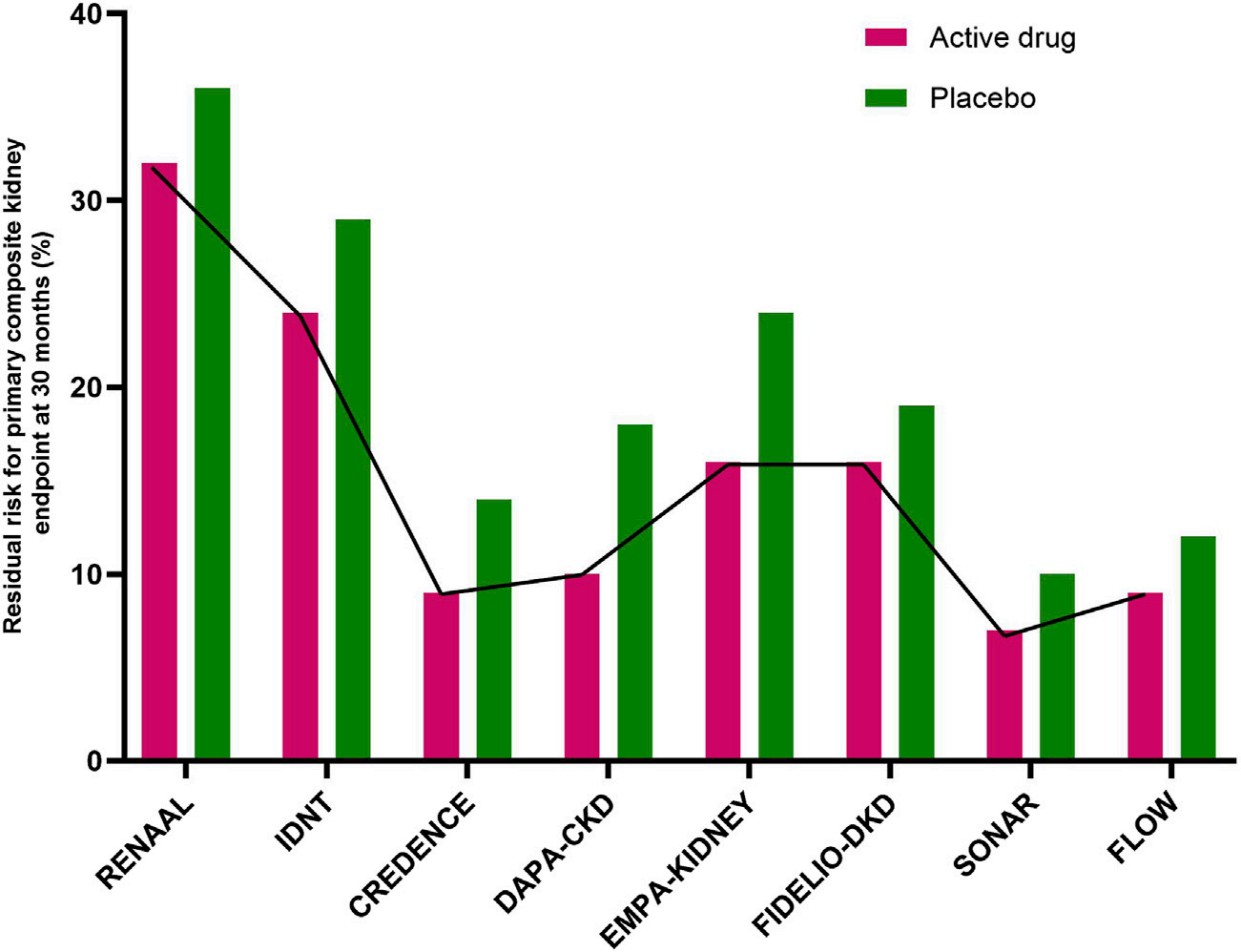
Residual risk for the primary composite kidney endpoint at 30 months in major randomized controlled trials. The active drugs were losartan (RENAAL trial), irbesartan (IDNT trial), canagliflozin (CREDENCE) trial), dapagliflozin (DAPA-CKD trial), empagliflozin (EMPA-KIDNEY trial), finerenone (FIDELIO-DKD trial), atrasentan (SONAR trial), and semaglutide (FLOW trial). In the reported studies (from CREDENCE to FLOW) the novel active drugs were added onto standard therapy with renin-angiotensin-aldosterone inhibitors). (CREDENCE, Canagliflozin and Renal Events in Diabetes with Established Nephropathy Clinical Evaluation; DAPA-CKD, Dapagliflozin and Prevention of Adverse Outcome in Chronic Kidney Disease; EMPA-KIDNEY, Study of Heart and Kidney Protection with Empagliflozin; FIDELIO-DKD, Finrenone in Reducing Kidney Failure and Disease Progression in Diabetic Kidney Disease; FLOW, Evaluate Renal Function with Semaglutide Once weekly; IDNT, Irbesartan Diabetic Nephropathy Trial; RENAAL, Reduction of Endpoints in NIDDM with the Angiotensin II Antagonist Losartan study; SONAR, Study of Diabetic Nephropathy with Atrasentan).

The pathogenesis of DKD, though initiated by a metabolic disturbance is perpetuated by multiple inter-linked pathways involving the hemodynamic, inflammatory, oxidative, and fibrotic systems (Tuttle et al., 2022). This Research Topic aims to understand these complex mechanisms, identify newer therapeutic targets, and test these agents in well designed clinical trials. Eight articles were published in this Research Topic [retrospective studies (2), animal studies (2), meta-analysis (2), clinical trial (1), and in-depth review (1)]. The in-depth review by Zhang et al. summarizes the proposed mechanisms of albuminuria, the current progress, and the future directions of therapeutic avenues in DKD.

Current practice guidelines recommend withholding SGLT-2i during critical illness requiring hospitalization (“sick day rules”) (Watson et al., 2023). However, in a multicenter retrospective cohort study of adults hospitalized with acute heart failure associated AKI, exposure to SGLT-2i during AKI was associated with no difference in time to renal recovery, but a lower 30-day mortality (Aklilu et al., 2023). Whether these findings can be generalized to all other etiologies of AKI is unknown. In this Research Topic, Alcantar-Vallin et al. explore the role of continuing or initiating SGLT-2i during an AKI episode. This was a retrospective cohort study involving 374 patients hospitalized with AKI, of whom 58 received SGLT-2i treatment during the hospitalization. The authors found that SGLT-2i use was not associated with risk of major adverse kidney events but was associated with a reduced risk of mortality (OR 0.08, 95% CI 0.01–0.64).

In the FLOW trial, semaglutide, a glucagon like peptide-1 receptor agonist (GLP-1RA) was shown to decrease clinically relevant kidney outcomes and death from CV causes in patients with type 2 diabetes and CKD (Perkovic et al., 2024). In a *post hoc* analysis of this trial, the benefits of semaglutide in reducing kidney outcomes were found to be consistent in participants with and without SGLT-2i use (Mann et al., 2024). In this Research Topic Cao et al. carried out a head-to-head comparison of GLP-1RA and SGLT-2i in patients with DKD. This was a randomized, open-label, non-inferiority trial. A total of 106 patients with type 2 diabetes and chronic kidney disease (CKD) were randomized 1:1 to receive either polyethylene glycol loxenatide (PEG-Loxe) 0.2 mg subcutaneous once weekly or dapagliflozin 10 mg orally once daily for 24 weeks. The primary outcome was change in urine albumin creatinine ratio (UACR) from baseline to 24 weeks. The authors showed that following 24 weeks of treatment there was no difference in change in UACR between the two groups (mean intergroup difference 2.6% (95% CI, -5.3 to -10.4%, p=0.336).

Diabetes is a state of oxidative stress and inflammatory burden, which contributes to complications including diabetic nephropathy. The heightened oxidative stress in diabetes is multi-factorial, such as, activation of polyol and hexosamine pathways, accumulation of glycolysis intermediates, activation of protein kinase C, mitochondrial dysfunction, etc., (Jin et al., 2023). There is a strong mutually enhancing interaction between inflammation and oxidative stress. Pro-inflammatory cytokines and chemokines activate reactive oxygen species (ROS) leading to macrophage activation, resulting in more oxidative stress. In return, ROS induce the expression of pro-inflammatory cytokines by activating transcription factors like nuclear factor-kappa B (NF-kB) and activator protein-1 (AP-1) (Caturano et al., 2023). Hence, addressing oxidative stress and inflammation as potential therapeutic targets for DKD has gained greater significance (Winiarska et al., 2021). In recent years, Chinese herbal medicines have been reported to attenuate progression of DKD by their anti-inflammatory and anti-oxidative properties (Zhong et al., 2015). Chen et al. identified 16 potentially renoprotective Chinese herbal medicines for DKD (PRCHMDKD) which were known to target multiple oxidative stress pathways. The authors then retrospectively analyzed the National Health Insurance Database of Taiwan from 2000 to 2017. They identified 43,480 PRCHMDKD users with DKD and 1,442 PRCHMDKD users with advanced DKD who were matched with an equal number of non-users using a nearest neighbour approach. PRCHMDKD use was associated with reduction in adjusted hazard ratios for kidney failure, all-cause-and CV mortality in the overall and advanced DKD population.

Huangkui capsule (HKC) is a traditional Chinese medicine which is currently approved for treatment of CKD in China. It is made from an ethanol extract of *Abelmoschus Manihot* flowers (Li et al., 2021). In a multicentre, double blind, placebo-controlled randomized clinical trial (RCT) conducted in China, the combination of irbesartan and HKC was found to significantly reduce albuminuria at 24 weeks as compared to irbesartan alone (Zhao et al., 2022). In this Research Topic, Tan et al. conducted a systematic review and meta-analysis of English and Chinese databases, including RCTs comparing HKC combined with RAASi versus RAASi alone in patients with DKD. This meta-analysis included 32 RCTs with a total of 2,881 patients with DKD. The authors showed that the combination of HKC with RAASi was more effective in reducing 24-hour urine protein, urine albumin excretion rate, UACR, serum creatinine, and blood urea nitrogen as compared to RAASi alone.

The primary pharmacologic metabolites in HKC have been identified as total flavonoids of A. Manihot (TFA). However, the mechanism by which these metabolites decrease albuminuria is unknown. Diao et al. carried out a comprehensive analysis of the primary flavonoid metabolites and their fates in db/db mice treated with HKC. A total of 7 flavonoid prototypes and 38 metabolites were identified in the serum, kidney, and urine of db/db mice. The flavonoid metabolite with the highest exposure in serum and kidney samples was quercetin monoglucuronide sulfate.

*Ophiocordyceps sinensis* (OS), popularly known as caterpillar mushroom is a medicinal fungus found in India and China (Dai et al., 2024). Currently, there are six main OS preparations available as Chinese patent medicine products. Xue et al. conducted an umbrella review of systematic reviews and Bayesian network meta-analysis to study the efficacy and safety of OS preparations combined with RAASi. A total of 157 RCTs involving 13,143 participants were included in the meta-analysis. The authors showed that addition of OS preparations to RAASi was more effective in reducing 24-hour urine protein levels as compared to RAASi alone. Of the 4 preparations studied, Jinshuibao capsules were found to be most effective.

Recent studies have shown that renal tubular injury in DKD is mediated by lipid accumulation via CD-36 and inflammation via NLRP3 inflammasome pathways (Qiu and Tang, 2016; Yang et al., 2017). Astragaloside IV (AS-IV) has been found to effectively suppress oxidative stress and fibrosis through downregulation of CD-36 in glomerular mesangial and myocardial cells. However, the impact of AS-IV on renal tubular injury in DKD rat models is unknown. In this Research Topic, Li et al. showed that administration of AS-IV reduced fasting blood glucose, UACR and 24-hour urine protein excretion in DKD rats. It also mitigated renal tubular injury and supressed expression of CD-36, NLRP3 and other inflammatory biomarkers. *In vitro* experiments using palmitic acid-induced HK cells also showed decrease in CD-36 expression, lipid accumulation and NLPRP3 activation with AS-IV treatment.

In conclusion, the articles in this Research Topic open out new therapeutic avenues in the management of DKD. With a rise in global prevalence of diabetes, the prevalence of DKD is expected to double over the next 20 years (Chen, 2022). This Research Topic exemplifies the urgent need to conduct more active research in this field targeting multiple pathogenic pathways.

## References

[B1] Aklilu A. M. Kumar S. Yamamoto Y. Moledina D. G. Sinha F. Testani J. M. (2023). Outcomes associated with sodium-glucose cotransporter-2 inhibitor use in acute heart failure hospitalizations complicated by AKI. Kidney360 4 (10), 1371–1381. 10.34067/kid.0000000000000250 37644648 PMC10615381

[B2] Alicic R. Z. Rooney M. T. Tuttle K. R. (2017). Diabetic kidney disease: challenges, progress, and possibilities. Clin. J. Am. Soc. Nephrol. 12 (12), 2032–2045. 10.2215/CJN.11491116 28522654 PMC5718284

[B3] Bakris G. L. Agarwal R. Anker S. D. Pitt B. Ruilope L. M. Rossing P. (2020). Effect of finerenone on chronic kidney disease outcomes in type 2 diabetes. N. Engl. J. Med. 383 (23), 2219–2229. 10.1056/NEJMoa2025845 33264825

[B4] Brenner B. M. Cooper M. E. Zeeuw D. d. Keane W. F. Mitch W. E. Parving H.-H. (2001). Effects of losartan on renal and cardiovascular outcomes in patients with type 2 diabetes and nephropathy. N. Engl. J. Med. 345 (12), 861–869. 10.1056/NEJMoa011161 11565518

[B5] Caturano A. D’Angelo M. Mormone A. Russo V. Mollica M. P. Salvatore T. (2023). Oxidative stress in type 2 diabetes: impacts from pathogenesis to lifestyle modifications. Curr. Issues Mol. Biol. 45 (8), 6651–6666. 10.3390/cimb45080420 37623239 PMC10453126

[B6] Chen J. (2022). “Diabetic kidney disease: scope of the problem,” in Diabetes and kidney disease. Editors Lerma, E. V. Batuman V. (Cham: Springer International Publishing), 37–47.

[B7] Dai Y. Chen S. Wang Y. Wang Y. Yang Z. Yu H. (2024). Molecular phylogenetics of the Ophiocordyceps sinensis-species complex lineage (Ascomycota, Hypocreales), with the discovery of new species and predictions of species distribution. IMA Fungus 15 (1), 2. 10.1186/s43008-023-00131-8 38336758 PMC10858606

[B8] Heerspink H. J. L. Parving H. H. Andress D. L. Bakris G. Correa-Rotter R. Hou F. F. (2019). Atrasentan and renal events in patients with type 2 diabetes and chronic kidney disease (SONAR): a double-blind, randomised, placebo-controlled trial. Lancet 393 (10184), 1937–1947. 10.1016/s0140-6736(19)30772-x 30995972

[B9] Heerspink H. J. L. Stefánsson B. V. Correa-Rotter R. Chertow G. M. Greene T. Hou F.-F. (2020). Dapagliflozin in patients with chronic kidney disease. N. Engl. J. Med. 383 (15), 1436–1446. 10.1056/NEJMoa2024816 32970396

[B10] Jin Q. Liu T. Qiao Y. Liu D. Yang L. Mao H. (2023). Oxidative stress and inflammation in diabetic nephropathy: role of polyphenols. Front. Immunol. 14, 1185317. 10.3389/fimmu.2023.1185317 37545494 PMC10401049

[B11] Lewis E. J. Hunsicker L. G. Clarke W. R. Berl T. Pohl M. A. Lewis J. B. (2001). Renoprotective effect of the angiotensin-receptor antagonist irbesartan in patients with nephropathy due to type 2 diabetes. N. Engl. J. Med. 345 (12), 851–860. 10.1056/NEJMoa011303 11565517

[B12] Li N. Tang H. Wu L. Ge H. Wang Y. Yu H. (2021). Chemical constituents, clinical efficacy and molecular mechanisms of the ethanol extract of Abelmoschus manihot flowers in treatment of kidney diseases. Phytother. Res. 35 (1), 198–206. 10.1002/ptr.6818 32716080 PMC7891592

[B13] Mann J. F. E. Rossing P. Bakris G. Belmar N. Bosch-Traberg H. Busch R. (2024). Effects of semaglutide with and without concomitant SGLT2 inhibitor use in participants with type 2 diabetes and chronic kidney disease in the FLOW trial. Nat. Med. 30 (10), 2849–2856. 10.1038/s41591-024-03133-0 38914124 PMC11485243

[B14] Perkovic V. Jardine M. J. Neal B. Bompoint S. Heerspink H. J. L. Charytan D. M. (2019). Canagliflozin and renal outcomes in type 2 diabetes and nephropathy. N. Engl. J. Med. 380 (24), 2295–2306. 10.1056/NEJMoa1811744 30990260

[B15] Perkovic V. Tuttle K. R. Rossing P. Mahaffey K. W. Mann J. F. E. Bakris G. (2024). Effects of semaglutide on chronic kidney disease in patients with type 2 diabetes. N. Engl. J. Med. 391 (2), 109–121. 10.1056/NEJMoa2403347 38785209

[B16] Qiu Y. Y. Tang L. Q. (2016). Roles of the NLRP3 inflammasome in the pathogenesis of diabetic nephropathy. Pharmacol. Res. 114, 251–264. 10.1016/j.phrs.2016.11.004 27826011

[B17] The EMPA-KIDNEY Collaborative Group Herrington W. G. Staplin N. Wanner C. Green J. B. Hauske S. J. Emberson J. R. (2023). Empagliflozin in patients with chronic kidney disease. N. Engl. J. Med. 388 (2), 117–127. 10.1056/nejmoa2204233 36331190 PMC7614055

[B18] Tuttle K. R. Agarwal R. Alpers C. E. Bakris G. L. Brosius F. C. Kolkhof P. (2022). Molecular mechanisms and therapeutic targets for diabetic kidney disease. Kidney Int. 102 (2), 248–260. 10.1016/j.kint.2022.05.012 35661785

[B19] Watson K. E. Dhaliwal K. Robertshaw S. Verdin N. Benterud E. Lamont N. (2023). Consensus recommendations for sick day medication guidance for people with diabetes, kidney, or cardiovascular disease: a modified delphi process. Am. J. Kidney Dis. 81 (5), 564–574. 10.1053/j.ajkd.2022.10.012 36470530

[B20] Winiarska A. Knysak M. Nabrdalik K. Gumprecht J. Stompór T. (2021). Inflammation and oxidative stress in diabetic kidney disease: the targets for SGLT2 inhibitors and GLP-1 receptor agonists. Int. J. Mol. Sci. 22 (19), 10822. 10.3390/ijms221910822 34639160 PMC8509708

[B21] Yang X. Okamura D. M. Lu X. Chen Y. Moorhead J. Varghese Z. (2017). CD36 in chronic kidney disease: novel insights and therapeutic opportunities. Nat. Rev. Nephrol. 13 (12), 769–781. 10.1038/nrneph.2017.126 28919632

[B22] Zhao J. Tostivint I. Xu L. Huang J. Gambotti L. Boffa J. J. (2022). Efficacy of combined Abelmoschus manihot and irbesartan for reduction of albuminuria in patients with type 2 diabetes and diabetic kidney disease: a multicenter randomized double-blind parallel controlled clinical trial. Diabetes Care 45 (7), e113–e115. 10.2337/dc22-0607 35613364 PMC9274216

[B23] Zhong Y. Menon M. C. Deng Y. Chen Y. He J. C. (2015). Recent advances in traditional Chinese medicine for kidney disease. Am. J. Kidney Dis. 66 (3), 513–522. 10.1053/j.ajkd.2015.04.013 26015275

